# Calcitic-based stones protection by a low-fluorine modified methacrylic coating

**DOI:** 10.1007/s11356-021-15515-9

**Published:** 2021-07-26

**Authors:** Eleonora Pargoletti, Valeria Comite, Paola Fermo, Valentina Sabatini, Luisa Annunziata, Marco Aldo Ortenzi, Hermes Farina, Giuseppe Cappelletti

**Affiliations:** 1 Dipartimento di Chimica, Università degli Studi di Milano, via Golgi 19, 20133 Milano, Italy; 2 Consorzio Interuniversitario Nazionale per la Scienza e Tecnologia dei Materiali (INSTM), via Giusti 9, 50121 Firenze, Italy; 3 CRC Materiali Polimerici “LaMPo”, Dipartimento di Chimica, Università degli Studi di Milano, Via Golgi 19, 20133 Milan, Italy

**Keywords:** Protective coatings, Fluorinated acrylic polymers, Ion chromatography, Cultural heritage, UV stability, Outdoor exposure, Durability

## Abstract

Atmospheric pollutants, such as NO_x_, SO_2_, and particulate matter, together with water percolation inside the stone pores, represent the main causes of cultural heritage decay. In order to avoid these undesired phenomena, the application of protective coatings represents a reliable solution. In this context, the present study focused on the synthesis of low-fluorine content methacrylic-based (MMA) polymeric resins characterized by seven F atoms (namely F7 monomer) in the lateral chains. Four different percentages (1.0, 2.5, 5.0, and 10.0%) of the present monomer were adopted to obtain a final polymeric structure showing the desired hydrophobicity, processability, and structural and thermal stability (even after accelerated UV aging tests). MMA_F7(1.0) seemed to be the optimal one; therefore, it was further applied onto Candoglia marble. Specifically, the treated substrates showed good surface hydrophobicity, water repellency, and water vapor transpirability. No color variation was observed even after a 1.5-year exposure in a real polluted environment (Monza Cathedral). Interestingly, the application of this coating hindered the atmospheric nitrates penetration inside the stones and, at the same time, it limited the sulfates (gypsum) formation, thus revealing a very promising marbles protection resin.

**Supplementary Information:**

The online version contains supplementary material available at 10.1007/s11356-021-15515-9.

## Introduction

In recent decades, the achievement of a certain degree of surface hydrophobicity has been one of the main challenging issues to deal with especially in the field of cultural heritage protection (Mosquera et al. 2014; Pino et al. 2017; Aslanidou et al. 2018; Eyssautier-Chuine et al. 2018; Lettieri et al. 2018). Indeed, water percolation together with atmospheric pollution are the principal causes that lead to the irreversible deterioration of stone monuments (Varotsos et al. 2009; Charola 2018; Lettieri et al. 2018). Particularly calcitic-based substrates, such as Candoglia or Botticino marbles widely used for cultural heritage monuments, are among the materials that can be damaged by these external agents, therefore leading to their irremediable decay. Specifically, once water and harsh agents enter the stone, several physicochemical processes, e.g., carbonate dissolution (Piacenti 1994; Minto et al. 2018), freezing/thawing cycles (Ruedrich et al. 2011; Iñigo et al. 2013; Török and Szemerey-Kiss 2019), and/or crystallization/precipitation (Fernandes 2006) can occur, dramatically affecting the quality and properties of such building materials. For instance, the Milan Cathedral, one of the most famous monuments worldwide, is mainly composed by calcitic marble (80–85% CaCO_3_ and the 15–20% other minerals (Dino et al. 2019)). This important monument preservation against physicochemical deterioration induced by atmospheric pollution has to be guaranteed (Manoudis et al. 2009a; Goffredo 2013; Jroundi et al. 2017; Ruffolo and La Russa 2019).

Hence, since water penetration plays a key role in the stones damaging processes, the application of protective coatings does represent a pivotal point to guarantee the stone artifacts preservation. Any stone protective must pass muster, i.e., it should show good adhesion to the stone substrates, sufficient water repellency, and natural water vapor permeability. Besides, it should have good transparency, easiness of use, as well as durability over time without ruining the original substrate appearance (Dudley Cecil 1999; Manoudis et al. 2009a; Andreotti et al. 2018). In the recent decades, acrylic-based polymers have been already deeply investigated since they show good transparency, promising mechanical properties, they are cheap and very easy to be synthesized (Zhang et al. 2013; Chuang Ma et al. 2019; Artesani et al. 2020). However, they still show some important shortcomings: they revealed to be poorly durable, scarcely hydrophobic and they do not assure sufficient water vapor transpirability (Karapanagiotis et al. 2012). Moreover, acrylate-based polymers (such as polymethyl methacrylate, PMMA) exhibits poor photochemical stability thus resulting not suitable for durable applications, especially in real outdoor cases since cross-linking reactions and/or chain scission can occur (Bergamonti et al. 2018; Ntelia and Karapanagiotis 2020; Artesani et al. 2020). In the last decades, one of the most exploited acrylic-based resins has been Paraloid B72® easily synthesized via free radical polymerization, showing sufficient hydrophobicity and optical transparency. However, its main disadvantage lies in the labile hydrogen atom in alpha position to carbonyl group (in the acrylic moiety) which can be easily removed forming radicals able to start photochemical reactions, thus resulting in the coating degradation and yellowing (Ntelia and Karapanagiotis 2020).

In order to overcome such drawbacks, especially to increase the hydrophobicity degree, partially fluorinated acrylic polymers were adopted (Toniolo et al. 2002b; Castelvetro et al. 2002; Malshe and Sangaj 2005; Papadopoulou et al. 2010; Yao et al. 2014): for example, Alessandrini et al. (2000) corroborated the presence of fluorine always has, as expected, a positive influence on the protective behavior increasing the water repellency of the coated stones. Besides, Poli et al. (2004) investigated the introduction of fluorine atoms in the ester chains of methyl acrylate-based polymers resulting in a drastic increase of the stones water repellency; nevertheless, they also stressed the importance of deeply studying the interaction between the polymers themselves and the adopted stone materials, and not only the protective physicochemical features. Besides, despite the steps forward especially in terms of greater hydrophobicity, the same shortcomings hinted for polyacrylates, mainly concerning durability, are still under debate (Mazzola et al. 2003; Bergamonti et al. 2018; Raneri et al. 2018; Artesani et al. 2020). Hence, there is still an urgent need to develop highly performing protectives able to get over the technological problems for both polyacrylates and their fluoro-derivatives.

In this scenario, several previous works (Poli et al. 2004; Yao et al. 2014; Sabatini et al. 2018a, 2018b, 2019) have already highlighted the promising features of fluorinated methacrylic polymers, coupling the better hydrophobic features deriving from fluorine-based co-monomers with the intrinsic photo-chemical stability of polymethylmethacrylates. Especially, Sabatini et al. (2019) in a very recent study evidenced that the co-monomer based on methyl methacrylate (MMA) and 3,3,4,4,5,5,6,6,7,7,8,8,8-tridecafluoro-octyl methacrylate (POMA, 1% mol×mol^−1^) is a very good alternative showing sufficient water vapor permeability, limited water absorbed by capillarity without occluding marble pores, transparency, good photochemical stability, and easiness of removal. Thus, starting from these results, herein the same synthetic route was adopted to prepare a polymeric protective with improved properties minimizing the fluorine content, due to the high costs related to the synthesis and the use of fluorinated compounds (Malshe and Sangaj 2005). Specifically, instead of POMA, *1H*,*1H*-heptafluoro-n-butyl methacrylate (F7) was adopted at different concentrations. In order to assess the coatings efficiency, a deep physicochemical investigation on both pure polymers and Candoglia marble coated specimens was performed. In details, wettability features, water vapor permeability, and colorimetric assessment together with soluble salt evaluation inside marbles were also investigated after a 1.5-year outdoor exposure at Monza Cathedral, a typical polluted urban environment in the northern part of Italy.

## Material and methods

All the reagents and solvents (by Sigma-Aldrich) were of reagent-grade purity and used without purification. The adopted lithotype is Candoglia calcitic marble (80–85% CaCO_3_ and the 15–20% other minerals including epidote, quartz, barite, and Ba-feldspar (Dino et al. 2019)) that has a characteristic pinkish to grayish color and a coarse-grained texture (> 3 mm). Moreover, some centimeter-thick dark-greenish silicate layers (mainly represented by diopside and tremolite) characterize the texture of the marble. The stone porosity is less than 1% (Toniolo et al. 2002a; Villa et al. 2020). It has been largely used in local rural constructions and historical buildings (for example Monza Cathedral in Italy), but its most famous application has been for the Milan Cathedral construction (back to fourteenth century).

### Synthesis of MMA_F7 polymeric resins, their characterization, and aging tests

**Fig. 1:**
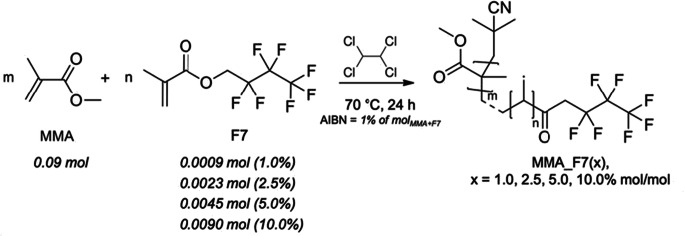
Adopted synthetic route together with the amounts used for each reagent

Starting from our previous works (Sabatini et al. 2018b, 2019), polymeric coatings based on methyl methacrylate (MMA) and *1H*,*1H*-heptafluoro-n-butyl methacrylate (F7) were synthesized via free radical polymerization, adopting F7/MMA at different molar percentages (i.e., 1.0, 2.5, 5.0, and 10.0%) (Pargoletti et al. 2020). Figure 1 shows the followed synthetic path. Specifically, in a 100-cm^3^ one-necked flask equipped with a magnetic stirring bar and a reflux condenser, the suitable amounts of MMA, F7, the initiator α,α’-azoisobutyronitrile (AIBN, mol_AIBN_ = 1% of mol_MMA+F7_) and tetrachloroethane as solvent (20 cm^3^) were added, and the flask was placed into a thermostatic oil bath under nitrogen atmosphere. The reaction was stirred at 70 °C for 24 h. The product was then precipitated using a large excess of cyclohexane, filtered, and then dried under vacuum at 40 °C overnight to eliminate traces of solvent and impurities.

The as-obtained polymeric materials were characterized by ^1^H-nuclear magnetic resonance (^1^H-NMR), Fourier transformed infrared spectroscopy (FT-IR), differential scanning calorimetry (DSC), and size exclusion chromatography (SEC), as already reported in our previous study (Sabatini et al. 2019). NMR spectra were collected at 25 °C with a BRUKER 400 MHz spectrometer. In this case, the samples were prepared dissolving 8–10 mg of polymer in 1 cm^3^ of CDCl_3_.

DSC analyses were conducted using a Mettler Toledo DSC1; the analyses were performed weighting 5–10 mg of the sample in a standard 40 μL aluminum pan, following the thermal program reported elsewhere (Sabatini et al. 2019). The first heating and cooling cycles were necessary to dry the samples and the glass transition temperatures (T_g_) were determined in the last heating cycle.

FT-IR spectrum was collected on a Spectrum 100 Perkin-Elmer spectrophotometer in attenuated total reflection (ATR) mode using a resolution of 4.0 and 256 scans, in a range of wavenumbers between 4000 and 400 cm^−1^.

The number-average molecular weight ($\overline{Mn}$) was investigated by size exclusion chromatography (SEC), using a refractive index (RI) detector and tetrahydrofuran as eluent. Molecular weight values are expressed in polystyrene (PS) equivalents.

To further study the wettability properties of the polymer itself, water contact angles (WCAs) using a Krüss Easydrop Instrument were determined. WCA values were obtained by depositing a drop (ca. 5 μL) of Milli-Q water on polymeric films (4 × 4 cm) obtained via solvent casting deposition. Particularly, samples were dissolved in chloroform and cast onto a polytetrafluoroethylene (PTFE) mold. All the data were averaged over at least ten measurements for every specimen to guarantee a statistical population.

Finally, in order to unravel the long-term properties of the as-prepared resin, a UV accelerated test was performed for 100 h, at 25 °C, 1 atm, relative humidity (RH % of ca. 40%) and with an Ultra Vitalux lamp characterized by a wavelength of 315–400 nm for UVA rays (UVA radiated power 13.6 W) and 280–315 nm for UVB ones (UVB radiated power 3.0 W), according to UNI 10925 2001 standard method (UNI 10925 2001). The lamp was placed at 40 cm above the samples.

### Coating application on Candoglia marble

Small blocks (about 2 cm × 2 cm × 2 cm or 5 cm × 5 cm × 1 cm) of Candoglia marble were polished with commercial grade diamond abrasive disks before any treatment (Fermo et al. 2014) and dried in oven at 60 °C for a week.

The application of the prepared polymer on marble surfaces was carried out by using an air-brush system (Asturo airbrush, 700 mm nozzle). The best promising MMA_F7 polymer was dissolved in a solution of dichloromethane/toluene (1/2.5 v/v) with a concentration of 4 mg mL^−1^. The quantity of the sprayed coating was kept constant at 6 mL every 4 cm^2^ of surface area by controlling also the air-brush spray pressure (2.5 bar). All the samples were dried under fumehood overnight. Each specimen was weighed before and after the deposition obtaining an average deposited amount equal to 18 mg for each sample (Pargoletti et al. 2020).

In order to investigate the possible chemical interaction between marble surface and polymeric resin, FT-IR analyses were carried out on bare, treated samples and on marble after having removed any coating trace (i.e., by immerging the 5 × 5 × 1 cm tile into 20 cm^3^ of dichloromethane/toluene (1/2.5 v/v) for 30 min, followed by a washing step with fresh water and a drying one in oven at 60 °C for 24 h) adopting a Spectrum 100 Perkin-Elmer spectrophotometer in attenuated total reflection (ATR) mode. Spectra were collected using a resolution of 4.0 and 64 scans, in a range of wavenumbers between 4000 and 400 cm^−1^.

### Physicochemical characterization of untreated and coated Candoglia marbles

As for bare polymeric films, WCA measurements on untreated and coated substrates were performed using a Krüss Easydrop instrument. A drop of 5 μL was gently placed on the surface; the drop profile was extrapolated using appropriate fitting functions (Circle and Tangent fitting methods) depending on the drop shapes. Measurements were repeated at least ten times to obtain a statistical population.

Colorimetric analyses were carried out directly on the different areas of the marble surface, before and after the coatings deposition, by means of a Konica Minolta CM 2300d portable spectrophotometer, referring to the CIELab diagram and to the UNI EN 15886 (UNI EN 15886 2010; Bergamonti et al. 2018; Pargoletti et al. 2020). The instrument was calibrated with its white reference (100% reflective) and zero calibration box (0% reference) in the 400–700 nm range. At least five measurements were performed on each area and the mean values of both L*, a*, b* and L*, C*, h* colorimetric parameters were reported. Indeed, the latter color space is similar to the former, but it describes color using cylindrical coordinates instead of rectangular ones. In this color space, L* indicates lightness, C* represents chroma, and h* is the hue angle. Chroma and hue are calculated from the a* and b*. According to the literature, no significant variation occurs when ΔE* < 5 (La Russa et al. 2012; Cappelletti et al. 2015).

Scanning electron microscopy (SEM) was carried out using a SEM Hitachi TM-1000 microscope. Elemental composition was obtained by means of Energy Dispersive X-ray spectroscopy (EDX) using Hitachi ED3000 spectrophotometer.

Capillary water absorption measurements were performed on both untreated and coated materials following the standard protocol UNI EN 15801 “Conservation of cultural property - test methods - determination of water absorption by capillarity” (UNI EN 2010; Cappelletti et al. 2015; Pino et al. 2017). The total amount of water absorbed (Q_tf_), the capillary absorption (CA) coefficient and the relative capillary index (IC_rel_, computed for both pre- and post-exposition) were determined, accordingly.

The water vapor permeability (WVP) tests were performed by means of a methodology described in the European Standard Norma EN 15803 (Manoudis et al. 2009b; UNI EN 2009; Pino et al. 2017) and the reduction of vapor permeability (RVP) was evaluated.

Moreover, to verify the homogeneity of the coated samples (roughness and thickness), the profile of the substrates surface was investigated by means of a contact profilometer (Bruker DektakXT), adopting a non-contact mode as reported elsewhere (Pargoletti et al. 2021).

### Outdoor exposure to assess coated marble durability

**Fig. 2:**
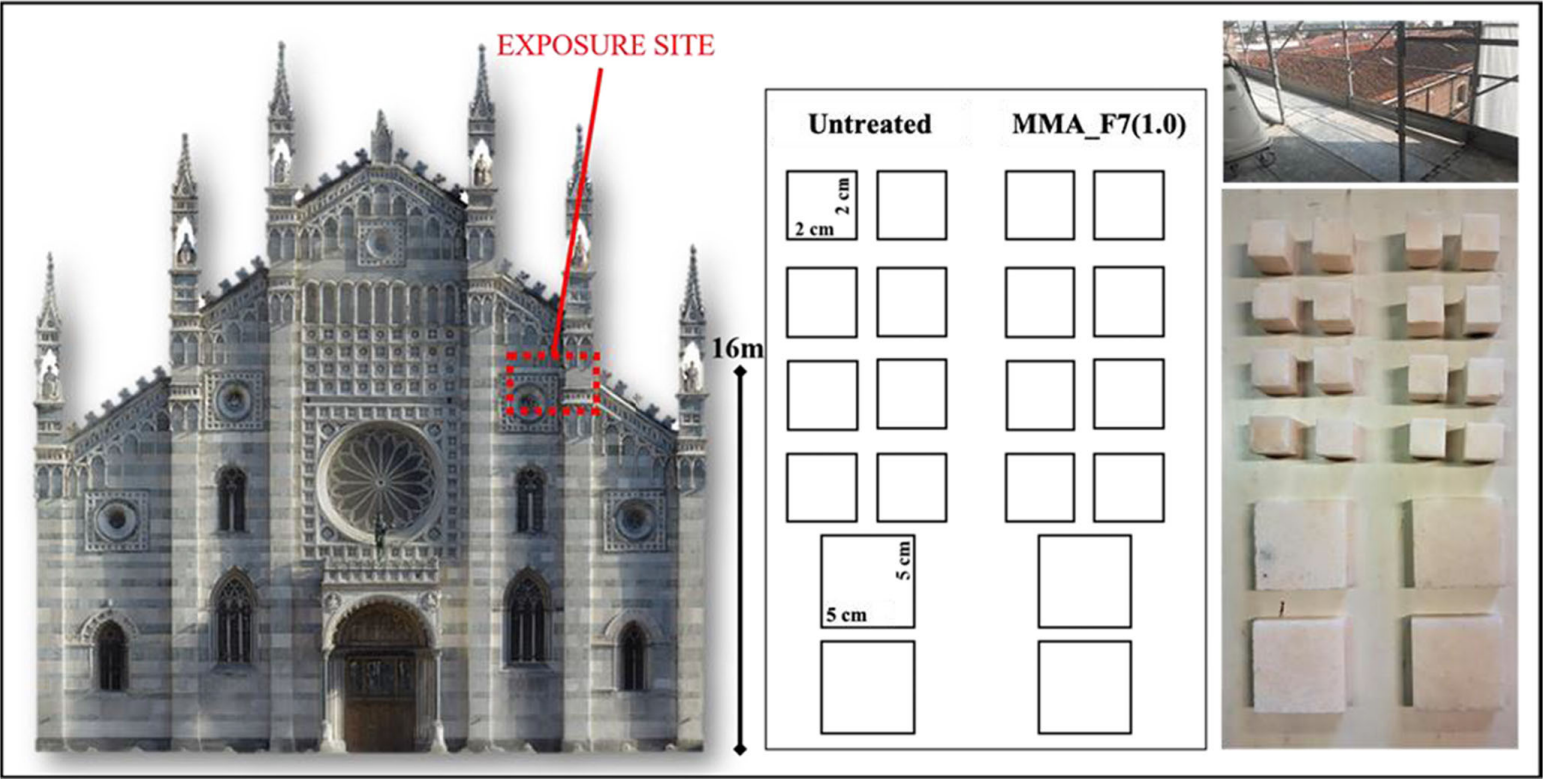
Exposure site at Monza Cathedral together with a schematic representation of the exposed samples

In order to assess the efficacy of the most promising polymeric coating in preventing marbles deterioration, both untreated and coated stones were exposed in a typical urban environment placed at the top of Monza Cathedral (see Fig. 2). The chosen site was at open air without any washout protection. The exposure test was carried out for 493 days (from 22 March 2019 to 28 July 2020). The wetting properties (WCA measurements and water absorption capillary studies) of the untreated and coated samples, together with the materials permeability and their surface color variation (CIELab method) were investigated after the outdoor exposure.

Moreover, ion chromatography (IC) was employed for the quantification of the main inorganic constituents of the deposits. About 2 mg of sample powder, collected according to our previous works (Pino et al. 2017; Sabatini et al. 2019), was placed in a test tube and treated with 10 mL of Milli-Q water. The solutions were put in an ultrasonic bath for 1 h, then centrifuged for 30 min and injected for IC analyses. Measurements of anionic (Cl^−^, NO_3_^−^, and SO_4_^2−^) species were carried out by using an ICS-1000 system equipped with a conductivity system detector. The analysis was carried out by an IonPac AS14A column (Dionex S.p.a., San Donato Milanese, Italy) using 8 mM Na_2_CO_3_/1 mM NaHCO_3_, flow rate of 1 cm^3^ min^-1^, using a conductivity system detector working with an anion self-regenerating suppressor ULTRA (ASRS-ULTRA) (Dionex S.p.a., San Donato Milanese, Italy). A blank sample consisting of a marble specimen, both not treated and not exposed, was also analyzed (namely absolute white). Anions concentrations determined in the blank sample (namely C) were subtracted from values obtained from the exposed ones, either untreated or treated.

## Results and discussion

### Polymer synthesis and their physicochemical characterization

**Fig. 3:**
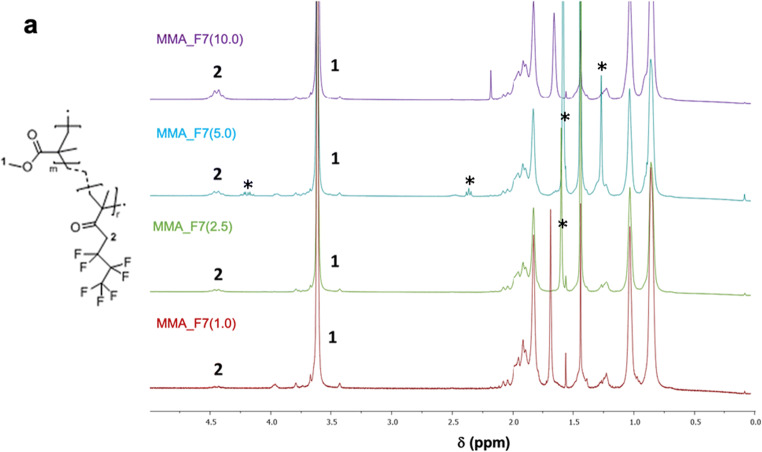
^1^H-NMR (proton nuclear magnetic resonance) spectra of all the synthesized MMA_F7 polymers. * = unidentified peaks

Proton nuclear magnetic resonance analysis (^1^H-NMR) has been performed to corroborate the effective synthesis accomplishment and the structure of the obtained MMA_F7-based polymers. The real amount of fluorine in samples was determined by considering the integral areas of peaks 1 and 2, highlighted in Fig. 3. As clearly visible in NMR spectra comparison (Fig. 3) and in Table S1, the experimental values of F7/MMA molar ratios are fully in accordance with the theoretical ones therefore evidencing the actual insertion of the fluorinated monomers into the MMA matrix.

Structural composition was also investigated by infrared spectroscopy. Figure S2 exhibits the FT-IR spectrum of MMA_F7(1.0) as representative sample. Notably, the typical peaks of methacrylate-based polymers, i.e., aliphatic -CH and -CH_2_ stretching at about 3100–2900 cm^−1^ (*a* in Fig. S2), the intense C=O stretching peak slightly above 1700 cm^−1^ (*b*), and the one at about 1100 cm^−1^ due to C-O(ester) stretching (*c*), are clearly observable. Furthermore, C-F stretching modes in the 1400–1000 cm^-1^ range is probably covered by the far more intense peak of C-O(ester) stretching one.

Furthermore, size exclusion chromatography (SEC) together with differential scanning calorimetry (DSC) measurements were carried out before and after an accelerated UV aging test to evaluate the polymers stability. As reported in Table 1, at the lowest fluorine contents (1.0–2.5%), the presence of the fluorinated groups seems to slow down the photodegradation process, which is still determined by the intrinsic reactivity of the alkyl group, as confirmed by previous research literature (Chiantore et al. 2000; Yao et al. 2014; Ruffolo and La Russa 2019; Huang et al. 2021). On the contrary, by increasing the fluorine content up to 10%, a certain degree of instability can be noticed: the number average molecular weight ($\overline{Mn}$) drastically rose up, as well as the size distribution (D) that remarkably changed alongside with the glass transition temperature (T_g_). In particular, this last parameter evidenced the insufficient photo-chemical stability of these high F-content polymers (especially of MMA_F7(10.0) that suffered from a T_g_ loss of about 20 °C). Indeed, having a glass transition temperature quite low (around 60 °C because of both the increased chain mobility and a higher presence of fluorine-based pendants), a possible chain degradation by a local temperature variation due to UV exposure could occur. As such, this phenomenon led to the cross-linking of the polymer structures, promoted by the radicals formed on the alkyl side groups (Chiantore et al. 2000; Castelvetro et al. 2002), resulting in a rise of the average molecular weight (from 18900 to 41400 for MMA_F7(10.0); see Table 1). Conversely, the T_g_ data of the polymers with 1.0–5.0% of F7 are relatively high, with MMA_F7(1.0) being the highest one (see Table 1 and Fig. S1).

**Table 1:** Size exclusion chromatography (SEC) data (number average molecular weight, $\overline{Mn}$ and molecular weight distribution, D), glass transition temperature (T_g_) and water contact angles (WCAs) of MMA_F7 polymers before and after the UV aging test

Samples	$\overline{Mn}$(Da)		D		T_g_ (°C)		WCA (°)			
							Air-side		Mold-side	
	UV aging									
	Pre	Post	Pre	Post	Pre	Post	Pre	Post	Pre	Post
MMA_F7(1.0)	28,500	27,000	3.8	3.2	94	n.d.	74 ± 2	73 ± 2	95 ± 5	93 ± 5
MMA_F7(2.5)	19,000	17,700	5.8	3.9	80	n.d.	71 ± 5	71 ± 2	96 ± 5	96 ± 3
MMA_F7(5.0)	20,900	22,300	4.1	3.3	78	98	80 ± 3	80 ± 2	106 ± 6	103 ± 9
MMA_F7(10.0)	18,900	41,400	3.7	3.1	64	42	84 ± 4	80 ± 7	114 ± 6	109 ± 6

Besides, after having deposited and let dry the polymeric materials in PTFE molds, the wetting properties of the obtained foils were investigated by water contact angle (WCA) measurements on both air and mold sides (Table 1). This test was also repeated after the UV aging test. The hydrophobic behavior seems to slightly enhance with the increasing of the fluorine content in accordance with the intrinsic character of F-containing groups (Yao et al. 2014). Indeed, fluorine-based molecules are characterized by scarce wettability and extremely low surface energy thanks to the high ionization potential of fluorine and its low polarizability. Moreover, as already studied in our previous works (Sabatini et al. 2018b, 2019), the WCA difference between the air and PTFE sides for all the polymer foils is above ca. 20° and it may be ascribable to the reorganization of the fluorinated groups of the polymeric chain during the solvent evaporation and to their high affinity with the hydrophobic PTFE mold surface. Indeed, this difference increased by rising the F-based monomer content. As far as it concerns the aging test, the WCAs did not vary so much evidencing the promising surface features stability even after an accelerated aging process.

### Application of polymeric coatings onto Candoglia marble and its durability upon outdoor exposure

By combining the results obtained through SEC, DSC, and after the aging test, MMA_F7(1.0) polymer was chosen to be applied onto Candoglia marble substrates, notwithstanding the lower WCA value achieved. Indeed, thanks to its greater photo-chemical stability and sufficient hydrophobic behavior, it has believed to be the optimal one for further application studies. In particular, its high T_g_ value can allow the possible user to apply it very easily. Also MMA_F7(2.5) and MMA_F7(5.0) were considered as suitable but they were not investigated due to a lower T_g_ and to the presence of few impurities detected via NMR (see highlighted peaks in Fig. 3) that might alter the results obtained during exposure.

**Fig. 4:**
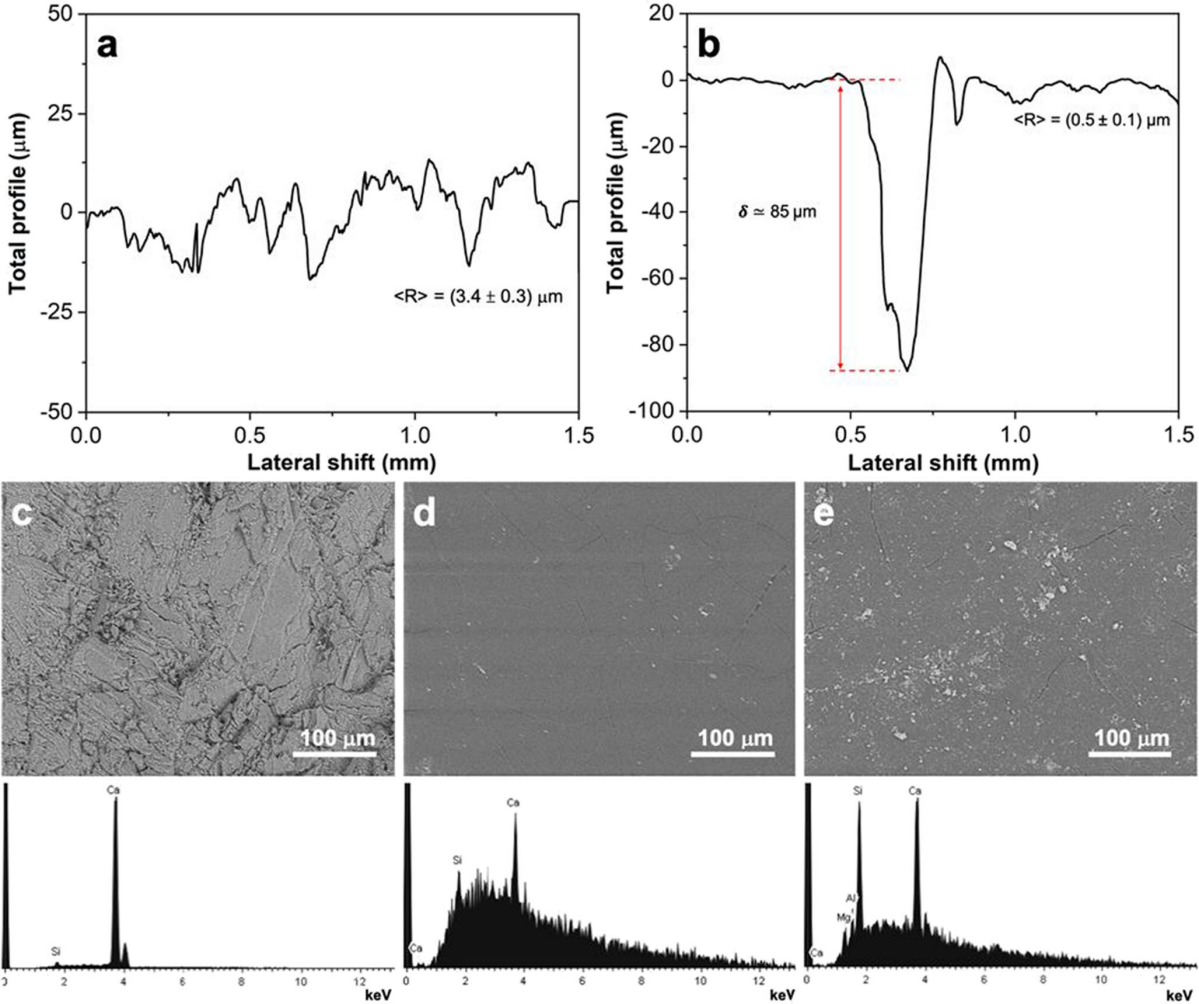
Topographic profile of **a** untreated C and **b** MMA_F7(1.0)@C. The roughness value (<R>) is reported as average value with the relative standard deviation. The coating thickness (highlighted by red arrow in **b**) was evaluated by scratching the film with a metallic pin. Scanning electron microscopy (SEM) images of **c** untreated marble, MMA_F7(1.0)@C, **d** before and **e** after the outdoor exposure together with the corresponding energy-dispersive X-ray spectroscopy (EDX) spectra

Hence, both thickness and roughness of untreated (Fig. 4a) and coated (Fig. 4b) samples have been investigated by means of profilometric measurements. Figure 4 b shows the total profile of the coated one in which a small scratch with a metallic pin was done to have an estimation of the coating thickness. The average width of the coated polymer is about 85 μm exhibiting a significant levelling effect after the deposition, since the marble surface roughness decreased from around 3.5 μm of untreated substrate to an average value of ca. 0.5 μm (see Fig. 4a, b and Table 2). This observation was further corroborated by SEM micrographs reported in Fig. 4 c and d, in which it is clearly visible that the surface texture of the pristine Candoglia was somewhat levelled by applying the polymeric protective. Furthermore, the effective presence of an organic matrix on the specimen surfaces was also confirmed by energy-dispersive X-ray (EDX) spectra (Fig. 4) since a broad background appeared in the case of coated samples.

**Table 2:** Comparison of water contact angles (WCAs), colorimetric variation (ΔE*), water capillary absorption by computing the final quantity of water absorbed (Q_ft_), the capillary absorption coefficient (CA) and the relative capillary index (IC_rel_), reduction percentage of vapor permeability (%RVP) relative to both bare and coated Candoglia marbles, before and after the outdoor exposure.

Samples	<R> (μm)	WCA (°)		ΔE*		Q_ft_ (mg cm^−2^)		CA (mg cm^−2^ s^−1/2^)		IC_rel_		% RVP	
		Outdoor exposure											
		Pre	Post	Pre	Post	Pre	Post	Pre	Post	Pre	Post	Pre	Post
C	(3.4 ± 0.3)	47 ± 4	71 ± 4	-	8.0	8.7	1.4	0.02	0	-	-	-	18
MMA_F7(1.0)@C	(0.5 ± 0.1)	76 ± 2	101 ± 3	2.6	1.9	2.0	1.0	0.01	0	0.10	0.50	39	19

Infrared spectroscopy was further adopted to assess the possible undesired formation of irreversible chemical interactions between the calcitic stone and the fluorinated polymeric resin: from the spectra comparison in Fig. S2, the main stretching modes (*d* at ca. 1400 cm^−1^, *e* at ca. 875 cm^−1^ and *f* at ca. 725 cm^−1^ relative to different asymmetric -CO_3_ stretching modes (Rodriguez-Blanco et al. 2011)) of calcium carbonate are almost covered by those characteristic of MMA_F7(1.0) polymer in the treated sample. Notably, no evidence of neither new peaks nor band shift was observed. On the contrary, after having washed out the resin from the marble surfaces, the resulted spectrum is identical to the pristine tile thus evidencing no permanent chemical interactions between the two materials and, therefore, avoiding any possible damage of the marble.

**Fig. 5:**
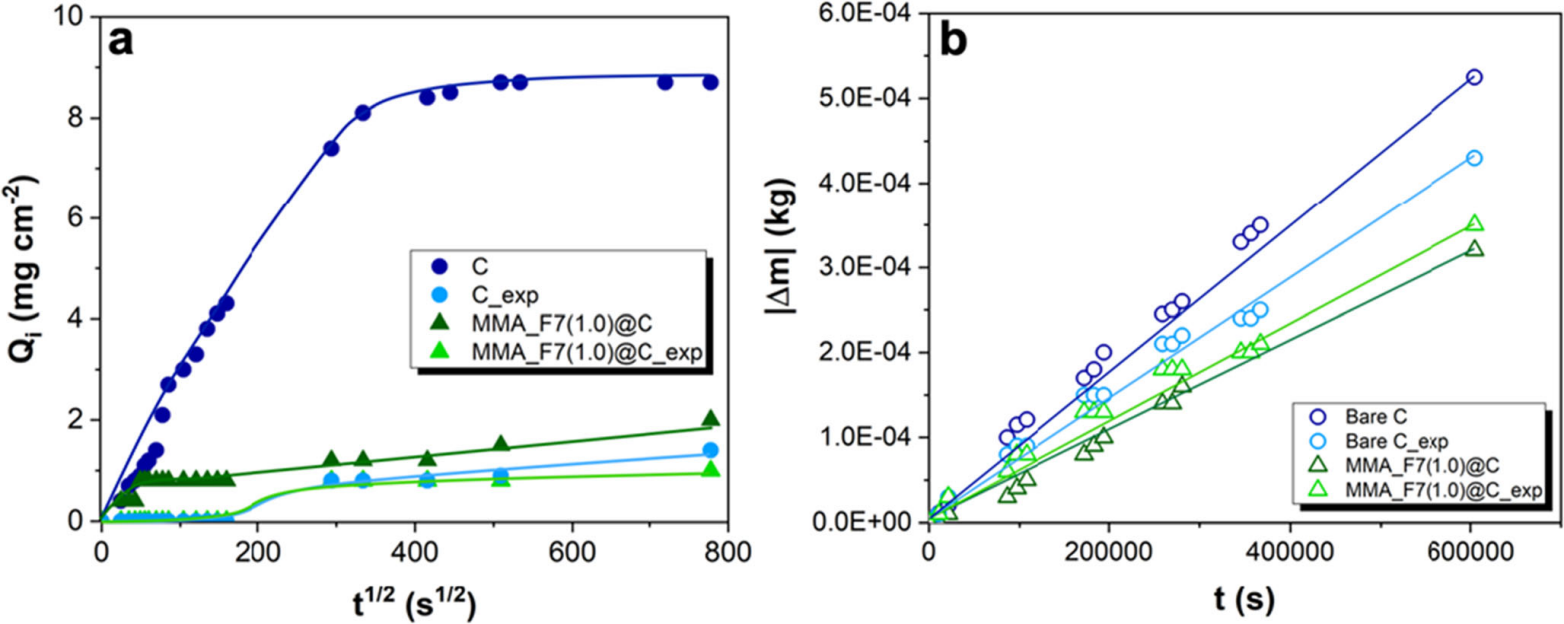
**a** Water absorption by capillarity and **b** water vapor transpirability by both untreated and MMA_F7(1.0)-treated Candoglia marbles, before and after outdoor exposure

In order to fully unveil the potential use of the so-synthesized polymer as protective resin for the preservation of stone artifacts, water contact angle measurements alongside with water capillary absorption, water vapor transpirability, and substrate color variation were deeply investigated. Regarding the actual wettability of the coated surfaces, a good improvement of the hydrophobic behavior was observed. Indeed, the average WCA increases of around 30° (Table 2), therefore allowing a reduction of the water percolation inside the material. This hindering was also corroborated by water absorption capillary tests reported in Fig. 5a. Specifically, the water amount absorbed at the end of the analysis (Q_tf_ in Table 2) was more than four times lower for the treated specimen with respect to the pristine one, together with a very low value of the relative capillary index that was around 1% (Table 2). In addition, the absorption capillary index (CA) halved after applying the MMA_F7(1.0) polymer, thus evidencing the promising protective features against water percolation. Besides, another parameter to consider is the water vapor permeability though stones pores. Indeed, a good coating should let the material hinder the water permeation but, at the same time, it should allow the stone breathing in order to avoid the possible cracks and mold/moss formation (De Buergo and Fort González 2001; Hens 2006; Kapridaki and Maravelaki-Kalaitzaki 2013). Figure 5 b displays the transpirability data of both untreated and coated samples, in terms of mass variation (Δm). The water vapor permeability reduction (RVP, Table 2) is around 40%, i.e., lower than the permitted threshold of 50%, thus guaranteeing a good stone breathability (UNI EN 2009).

Finally, a promising protective coating should also avoid any color variation when applied onto materials. Hence, CIELab colorimetric tests were performed to evaluate the ΔE* change that possibly indicates an undesired surface color modification. If ΔE* value was higher than 5, it would mean the material surface variation is visible also at naked eye (La Russa et al. 2012; Cappelletti et al. 2015). In the present case, the coatings application did not provoke any color change (see Table 2), thus underlining once more the efficacy of the synthesized MMA_F7(1.0) protective for Candoglia marble substrate. Moreover, by analyzing L*, C*, and h* colorimetric coordinates (see Table S2), we confirmed the previous outcomes since the hue variation (Δh*) is insignificant after the coating application, obtaining indeed a similar value of ΔE*.

Then, the further step forward was the exposure of both untreated and treated specimens in a real polluted environment. Since one the most famous historical buildings made by Candoglia marble is the Monza Cathedral, the studied samples were placed there on an open-air site without any washout protection. The exposure test was carried out for more than a year, from March 2019 to July 2020. After the exposure, either wettability/permeability features or colorimetric variations were investigated. As far as it concerns the WCAs and the absorption of water by capillarity, the degree of hydrophobicity was preserved or even enhanced probably due to hydrophobic dirt and impurities present on the materials surface after the prolonged exposure, as clearly evidenced from the amount of water absorbed at the final time (Q_tf_) and the relative capillary index (which slightly rose from 0.1 to 0.5). In order to confirm this hypothesis, SEM/EDX analyses were carried out on exposed samples. As noticeable in Fig. 4e, there are impurities on the coated stone most likely due to both external agents and air pollution. A further corroboration of this fact was obtained by means of CIELab test. Indeed, in particular for the untreated specimen, ΔE* parameter is quite high (ca. 8.0) with respect to the coated one (1.9, Table 2). This may indicate the possible presence of carbonaceous dirt or other contaminants on the surface, due to the open-air exposure, as also corroborated by the remarkable value of hue variation (Δh* greater than 2; Table S2).

Besides, the deposition of pollutants onto the exposed samples could have caused the partial occlusion of the stones pores, thus hindering the water vapor permeability. Instead, in this case study, the RVP percentage remained below the threshold of 50% (see Table 2), even after the outdoor exposure, revealing very promising also for its potential application in a real environment.

In order to better understand the polymeric coating efficiency towards the atmospheric pollution, IC analyses were performed. Indeed, the historical buildings exposed in a polluted environment undergo degradation processes due to the interaction between atmospheric gases and stone surfaces. It is recognized that the presence of nitrate and chloride anions are attributable to atmospheric deposition; sulfate, instead, is produced by the degradation of the stone calcium carbonate due to the interaction with atmospheric SO_2_, thus also generating the so-called black crusts which are mainly composed of newly formed gypsum crystals, salts, and atmospheric particles (Comite et al. 2020a, 2020b, 2020c; Fermo et al. 2020). Hence, in the present work, the main anions (chloride, sulfate, and nitrate ones) were analyzed on both untreated and treated Candoglia marbles exposed at the Monza Cathedral. For the sake of comparison, also untreated and unexposed samples (namely C) were considered and analyzed, as the reference. The average concentrations on eight different replicates are reported in Table 3. In the case of untreated sample, sulfates and nitrates data are of the same order of magnitude of those related to 1-year exposure tests already carried out in Milan metropolis on different marble substrates (i.e., Candoglia and Botticino ones) (Fermo et al. 2018; Sabatini et al. 2019). As such, the atmospheric pollution conditions of the previous works seem to be the same of this study. In particular, the presence of higher chloride values (Table 3), observed in the exposed treated sample (MMA_F7(1.0)@C_exp, i.e., ca. 420 ppm) compared to the untreated C (ca. 150 ppm), can be attributed to the use of dichloromethane adopted during the spraying deposition procedure. Conversely, as regards NO_3_^−^ ions (ca. 230 ppm for the untreated/exposed sample and about 690 ppm for the treated/exposed one), we observed a greater concentration for MMA_F7(1.0)@C_exp specimen, as already noticed in our previous works (Comite et al. 2017; Fermo et al. 2018; Sabatini et al. 2019). This phenomenon may be due to the ion penetration inside the lithotypes, which is instead hindered by the coating on the stones surfaces (Pargoletti et al. 2021). Then, in the case of sulfate concentration, the average value determined for C_exp specimen (ca. 3000 ppm; Table 3) is significantly higher (a 4-fold increase) with respect to MMA_F7(1.0)@C_exp (around 760 ppm), therefore showing the effectiveness of the polymeric coating in inhibiting the formation of sulfates. Therefore, comparing these performances (MMA_F7 onto Candoglia) with those previously assessed (Sabatini et al. 2019) in the case of MMA_POMA coatings (MMA modified by 3,3,4,5,6,7,8,8-tridecafluoro-octyl methacrylate onto Botticino), a similar protection efficiency is achieved. However, the novel synthesized MMA_F7 polymer could be more outstanding in terms of industrial cost production, due to the lower number of fluorine atoms in the polymeric chain considering the same concentration of monomer used.

**Table 3:** Ion chromatography anions concentrations relative to untreated-unexposed specimen (C), untreated (C_exp) and treated (MMA_F7(1.0)@C_exp) marbles exposed at the Monza Cathedral. For both untreated and treated marbles, 8 samples were considered and analyzed, and the average concentrations were reported

Sample	ppm		
	Cl^−^	NO_3_^−^	SO_4_^2−^
C	210 ± 30	80 ± 10	< 5
C_exp	150 ± 20	230 ± 40	3000 ± 190
MMA_F7(1.0)@C_exp	420 ± 30	690 ± 50	760 ± 40

## Conclusions

In the present work, low-fluorine content (F7) methacrylic-based polymers were synthesized aiming at applying them as Candoglia marble protective coatings. A deep physicochemical characterization of different F concentration inside the polymeric chain was carried out to unveil the optimal one in terms of surface hydrophobicity, structural, and thermal stability even after an accelerated UV aging test. Particularly, MMA_F7(1.0) was chosen and sprayed onto Candoglia substrates because it demonstrated to be the most promising balance between sufficient water repellency (WCA ca. 75°) and good processability (T_g_ value of about 90 °C)/photochemical stability.

The desired features of application easiness, color appearance preservation, significant reduction of water absorbed by capillarity (around 80%), and good stone breathability (below the threshold limit of 50%) were achieved. In addition, by exploiting infrared spectroscopy technique, we confirmed the non-occurrence of any chemical interaction between the calcitic marble and the fluorine-containing polymeric resin; this assures the possible complete cleaning of the marble surface allowing the restoration of the pristine substrate.

Moreover, by exposing both untreated and coated specimens in a real polluted environment, we successfully demonstrated the polymer efficacy against marble deterioration due to atmospheric pollution and salts crystallization inside the marble pores. Notably, a dual phenomenon occurred: in the case of nitrates, the MMA_F7(1.0) coating hindered their penetration inside the stone materials while, on the other hand, it inhibited the sulfate formation as expected. As such, the protectives shown herein resulted outstanding for the conservation of Candoglia marbles, paving the way for both their possible exploitation with other stone materials and the engineering of other polymeric compounds with enhanced hydrophobic features.

## Supplementary Information

Table S1: Comparison between theoretical and experimental (by ^1^H NMR) F7/MMA (%mol×mol^-1^); Table S2: Average CIELab (both L*, a*, b* and L*, C*, h* methods) coordinates variation before and after the outdoor exposure; Figure S1: Comparison of differential scanning calorimetry (DSC) outputs for all the investigated polymers; Figure S2: Comparison of FT-IR spectra relative to bare Candoglia marble (C), pure MMA_F7(1.0) resin, treated stones (MMA_F7(1.0)@C) and cleaned sample (C_cleaned). (DOCX 11603 kb)

## Data Availability

All data generated or analyzed during this study are included in this published article and in its supplementary information file.
