# Supplementary material for: Calcitic-based stones protection by a low-fluorine modified methacrylic coating
Source: Environ Sci Pollut Res Int. 2021 Jul 26;29(20):29455–66. doi: 10.1007/s11356-021-15515-9 (PMC9001578; doi:10.1007/s11356-021-15515-9)

**Supporting Information**

**Calcitic-based stones protection by a low-fluorine modified methacrylic coating**

Eleonora Pargoletti^a,b,*^, Valeria Comite^a,b^, Paola Fermo^a,b^, Valentina Sabatini^a,b^, Luisa Annunziata^a,c^, Marco Aldo Ortenzi^a,b,c^, Hermes Farina^a,b,c^ and Giuseppe Cappelletti^a,b,c^

^a^ Università degli Studi di Milano, Dipartimento di Chimica, via Golgi 19, 20133, Milano, Italy

b Consorzio Interuniversitario Nazionale per la Scienza e Tecnologia dei Materiali (INSTM), via Giusti 9, 50121, Firenze, Italy

c CRC Materiali Polimerici “LaMPo”, Dipartimento di Chimica, Università degli Studi di Milano, Via Golgi 19, 20133, Milan, Italy

e-mails: valeria.comite@unimi.it; paola.fermo@unimi.it; valentina.sabatini@unimi.it; luisa.annunziata@unimi.it; marco.ortenzi@unimi.it; hermes.farina@unimi.it; giuseppe.cappelletti@unimi.it

***** Corresponding author; Università degli Studi di Milano, Dipartimento di Chimica, via Golgi 19, 20133, Milano, Italy

**Table S1.** Comparison between theoretical and experimental (by ^1^H-NMR) F7/MMA (%mol/mol).

| **Sample** | **%mol/mol F7/MMA theoretical** | **%mol/mol F7/MMA ^1^H NMR** |
| --- | --- | --- |
| MMA_F7(1.0) | 1.0 | 0.7 |
| MMA_F7(2.5) | 2.5 | 2.3 |
| MMA_F7(5.0) | 5.0 | 4.5 |
| MMA_F7(10.0) | 10.0 | 9.2 |

**Table S2.** Average CIELab (both L*, a*, b* and L*, C*, h* methods) coordinates variation before and after the outdoor exposure.

| **Samples** | **Δ*L**** | | **Δ*a**** | | **Δ*b**** | | **Δ*C**** | | **Δ*h**** | | **Δ*E**** | |
| --- | --- | --- | --- | --- | --- | --- | --- | --- | --- | --- | --- | --- |
|  | **Pre** | **Post** | **Pre** | **Post** | **Pre** | **Post** | **Pre** | **Post** | **Pre** | **Post** | **Pre** | **Post** |
| C | − | -7.9 | − | 0.05 | − | 2.1 | − | 0.1 | − | 2.4 | − | 8.0 |
| MMA_F7(1.0)@C | 2.8 | 1.9 | -0.2 | -0.1 | -1.3 | -0.04 | -0.2 | 0.1 | -0.8 | 0.7 | 2.6 | 1.9 |

**Figure S1.** Comparison of Differential Scanning Calorimetry (DSC) outputs for all the investigated polymers.


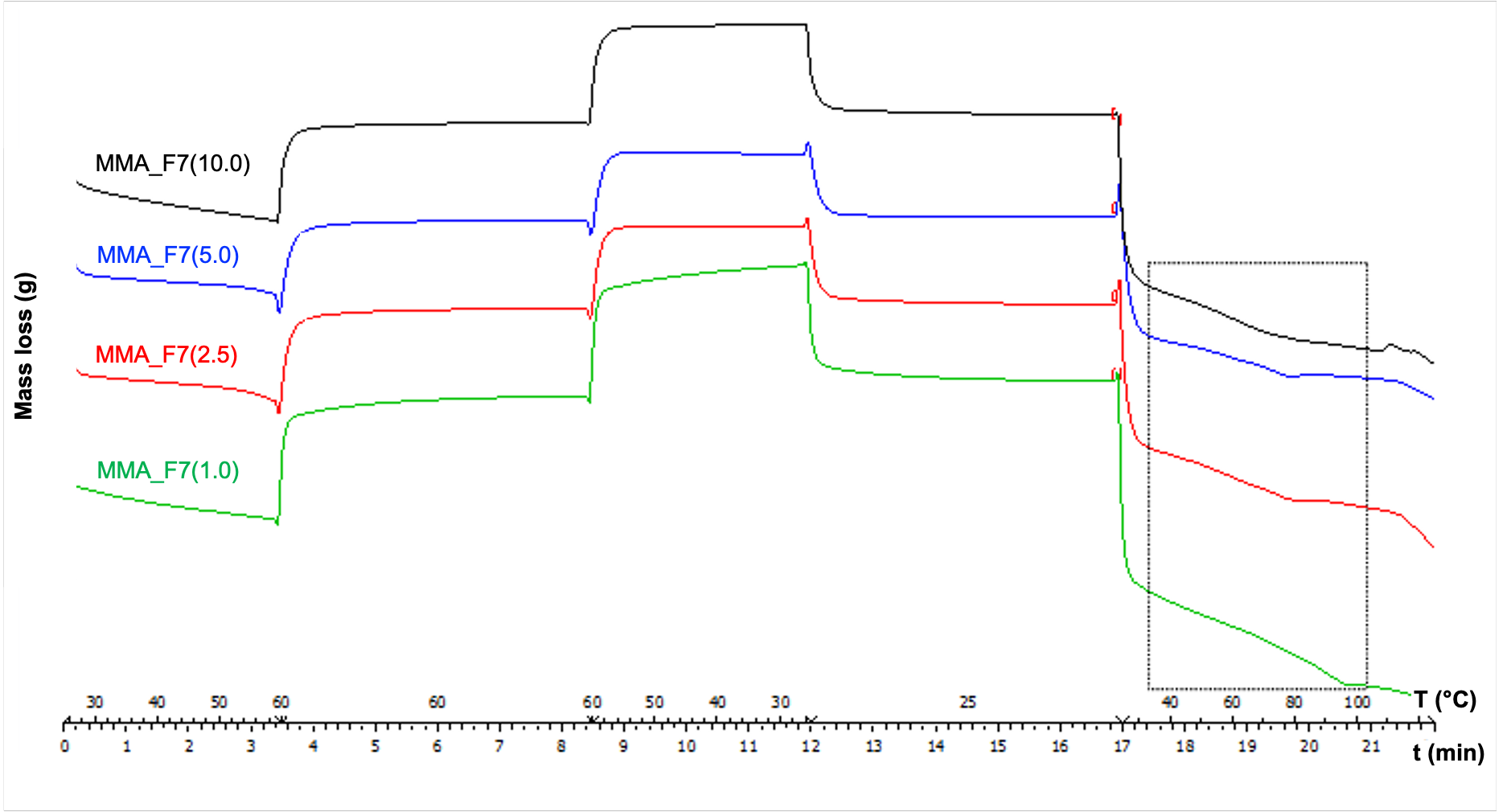


**Figure S2.** Comparison of FT-IR spectra relative to bare Candoglia marble (C), pure MMA_F7(1.0) resin, treated stones (MMA_F7(1.0)@C) and cleaned sample (C_Cleaned). The main peaks have been highlighted.


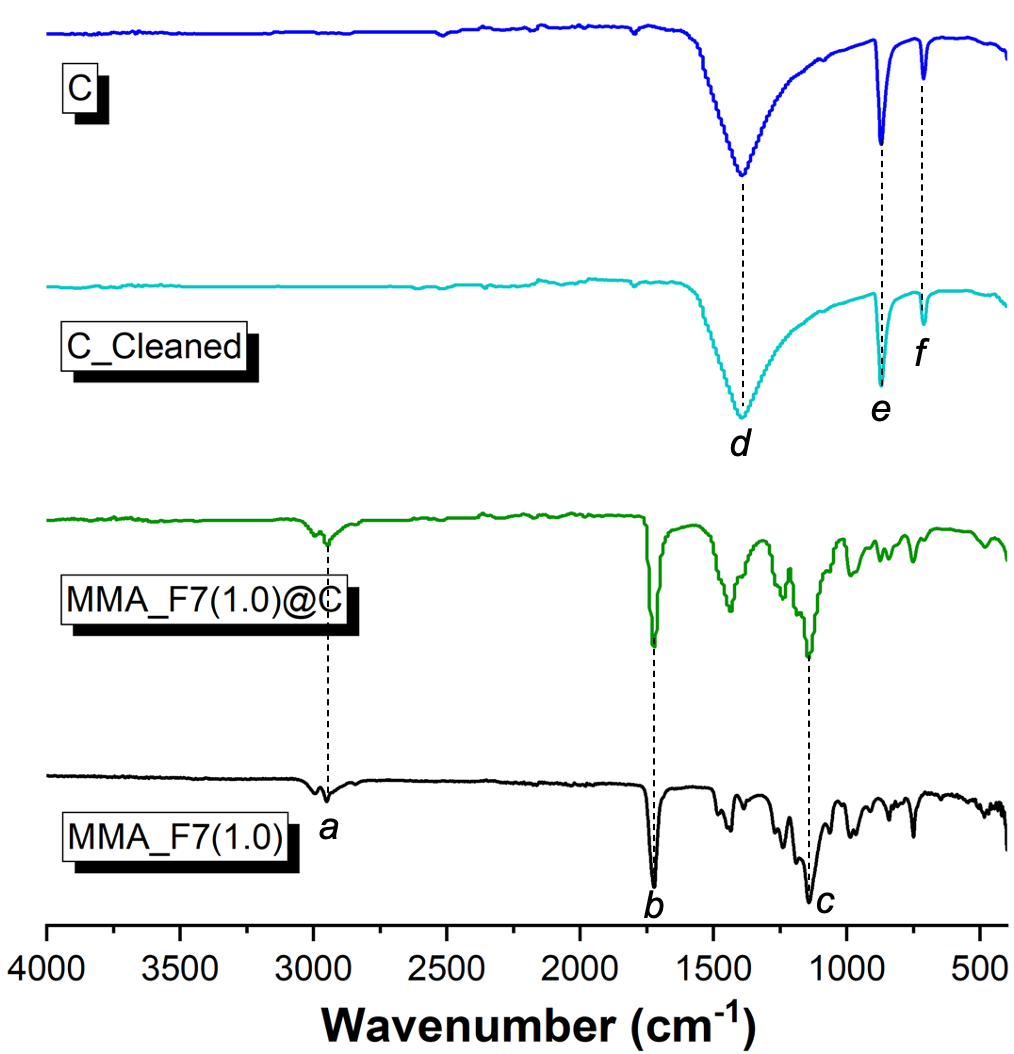

Supplement: Supplementary file 1 — Comparison between theoretical and experimental (by 1H NMR) F7/MMA (%mol×mol-1); Table S2: Average CIELab (both L*, a*, b* and L*, C*, h* methods) coordinates variation before and after the outdoor exposure; Figure S1: Comparison of differential scanning calorimetry (DSC) outputs for all the investigated polymers; Figure S2: Comparison of FT-IR spectra relative to bare Candoglia marble (C), pure MMA_F7(1.0) resin, treated stones (MMA_F7(1.0)@C) and cleaned sample (C_cleaned). (DOCX 11603 kb) [file 11356_2021_15515_MOESM1_ESM.docx]
